# Productivity costs of lifelong smoking—the Northern Finland Birth Cohort 1966 study

**DOI:** 10.1093/eurpub/ckae057

**Published:** 2024-03-29

**Authors:** Ina Rissanen, Iiro Nerg, Petteri Oura, Sanna Huikari, Marko Korhonen

**Affiliations:** Research Unit of Population Health, Faculty of Medicine, University of Oulu, Oulu, Finland; Medical Research Center Oulu, Oulu University Hospital and University of Oulu, Oulu, Finland; Julius Center for Health Sciences and Primary Care, University Medical Center Utrecht and Utrecht University, Utrecht, The Netherlands; Research Unit of Population Health, Faculty of Medicine, University of Oulu, Oulu, Finland; Department of Economics, Oulu Business School, University of Oulu, Oulu, Finland; Medical Research Center Oulu, Oulu University Hospital and University of Oulu, Oulu, Finland; Research Unit of Health Sciences and Technology, University of Oulu, Oulu, Finland; Department of Economics, Oulu Business School, University of Oulu, Oulu, Finland; Department of Economics, Oulu Business School, University of Oulu, Oulu, Finland

## Abstract

**Background:**

Smoking is one of the leading causes of impaired health and mortality. Loss of paid and unpaid work and replacements due to morbidity and mortality result in productivity costs. Our aim was to investigate the productivity costs of lifelong smoking trajectories and cumulative exposure using advanced human capital method (HCM) and friction cost method (FCM).

**Methods:**

Within the Northern Finland Birth Cohort 1966 (NFBC1966), 10 650 persons were followed from antenatal period to age 55 years. The life course of smoking behaviour was assessed with trajectory modelling and cumulative exposure with pack-years. Productivity costs were estimated with advanced HCM and FCM models by using detailed, national register-based data on care, disability, mortality, education, taxation, occupation and labour market. A two-part regression model was used to predict productivity costs associated with lifelong smoking and cumulative exposure.

**Results:**

Of the six distinct smoking trajectories, lifetime smokers had the highest productivity costs followed by late starters, late adult quitters, young adult quitters and youth smokers. Never-smokers had the lowest productivity costs. The higher the number of pack-years, the higher the productivity costs. Uniform patterns were found in both men and women and when estimated with HCM and FCM. The findings were independent of other health behaviours.

**Conclusions:**

Cumulative exposure to smoking is more crucial to productivity costs than starting or ending age of smoking. This suggests that the harmful effects of smoking depend on dose and duration of smoking and are irrespective of age when smoking occurred.

## Introduction

Smoking damages the general health, increases the risk of several chronic diseases, and is one of the leading causes of mortality worldwide.^1^^,^^2^ In spite of the progress in smoking reduction,^3^ a global analysis predicted that over one billion individuals will remain active smokers in 2025.^4^ The societal costs of smoking are huge, including direct health care costs and productivity losses associated with premature death, absenteeism and presenteeism. The total global economic cost of smoking is estimated at around US$1.85 trillion, or around 1.8% of global GDP.^5^ For individuals, smoking-related poor health affects work ability and lifetime earnings adversely. Since smoking is predicted to remain among the leading causes of morbidity and mortality, it is important to have precise estimates how smoking affects productivity.

Productivity costs are costs resulting from loss of paid and unpaid work, and replacement costs, due to morbidity and mortality. They are usually assessed in health economic evaluations with either human capital method (HCM) or friction cost method (FCM).^6–8^ The method to choose is an area of debate as both overestimation and underestimation may lead to erroneous decision-making.^7–10^ Recently, advanced HCM and FCM have been developed to tackle the shortcomings of traditional methods.^11^^,^^12^ Previous studies have estimated the productivity costs of smoking in various countries.^13–19^ For example, in the USA, estimated morbidity-related productivity costs attributable to cigarette smoking in 2018 dollars were $185 billion^15^ and mortality-related productivity costs $180 billion,^20^ showing enormous socioeconomic burden. A previous Finnish study showed that current smokers lost approximately 1.5 working years compared with never-smokers. Productivity costs due to premature deaths and disability accounted for 96% of the total health-related costs of smoking. However, nearly all previous productivity cost estimates have been done using traditional HCM and are probably overestimations of the true costs. Also, the previous productivity cost estimates are often imprecise due to the lack comprehensive data.

Initiation of smoking occurs commonly in adolescence or early adulthood.^2^^,^^21^ Previous studies have suggested that the duration of smoking (i.e. years smoked over the life course) may predict the risk of diseases more accurately than the intensity of smoking (i.e. cigarettes smoked per day) or pack-years (i.e. duration multiplied by intensity).^22–26^ Therefore, lifelong designs are needed to grasp the full effects of smoking on productivity costs, but such studies are scarce. Most previous studies assess smoking only cross-sectional ignoring the duration, timing, intensity and cumulative exposure.

In this study, we aim to investigate the productivity costs of smoking using a life-course design and advanced HCM and FCM, which allows us to estimate the costs more precisely. We use data from the NFBC1966 which is a population-based, unselected birth cohort. We use detailed and accurate register-based data and apply state-of-the-art productivity cost analyses controlling for occupation-specific costs, macroeconomic conditions, vacancy chains and disability conditions. We assess smoking using lifelong smoking trajectories and cumulative exposure. This study fills the important gap in the literature by providing most precisely quantified effect of smoking on productivity costs. In addition, we do not only compare smokers and non-smokers, we but also reveal evidence on productivity losses of former smokers, and of different starting and ending ages.

## Methods

We estimated HCM and FCM productivity costs of smoking trajectories and smoked pack-years in the NFBC1966 population, which is a non-selective, prospective, population-based birth cohort followed-up since the mid-pregnancy.^27^^,^^28^ The cohort is based on 12 058 alive-born children whose expected birth date was in 1966, representing 96% of children born in Finnish Provinces of Lapland and Oulu in 1966. The current study sample consisted of 10 650 persons who were alive and living in Finland at the age of 14 years, answered at least 10 questions in the postal questionnaire at the age of 14, and did not withdraw their consent in later follow-ups.

Smoking habits of cohort members were asked in questionnaires at ages 14, 31 and 46 years. The smoking status assessment and categorization is described in detail previously.^29^ The binary smoking status of each year in the persons’ life was used in the trajectory model. Smoked pack-years by age 46 were calculated based on information on smoking starting and ending ages and the question ‘How much per day do you usually smoke now or smoked before you gave up smoking?’.^30^ In the analyses, pack-years were categorized into ‘zero’, ‘under five’, ‘under 15’, ‘under 30’ and ‘over 30’ based on the distribution of the study population.

Covariates of the association between smoking and productivity costs included educational level, leisure-time physical activity, BMI, alcohol consumption, hypertension and hypercholesterolemia. Information on covariates was collected from follow-up questionnaires, clinical examinations and national registers at age of 46. Collection of covariates is described in Supplementary table S1. All analyses were stratified by sex (women/men) which was assigned at birth based on the visible external anatomy of a newborn.

Individual productivity costs were estimated with advanced HCM and FCM as described in our previous study.^12^ The population-level annual data, used in advanced HCM and FCM, was collected as described in the Supplementary table S2. In brief, data of death notes, including cause and date of death, were collected from Statistics Finland until February 2022. Data on sick leaves and disability pensions until the end of 2019 were collected from the registers of social insurance institution and Finnish Center for Pensions. The register of The Finnish Tax Administration was used to obtain individual-level data on the mean annual gross income of cohort members between 1995 and 2016.

In HCM, the productivity costs were calculated as days absent from work because of sick leave, disability pension or death until the statutory retirement age, multiplied with the value of daily production each year. In the advanced model, the median wage of each occupational class in Finnish population, stratified by sex, was used to monetarize the value of production. Furthermore, the model took future labor force participation rates into account.^11^^,^^12^

In FCM, the productivity costs were evaluated as length of the absence from work up to an estimated friction period, multiplied with the value of daily production. The friction period was annually estimated to be the occupation-specific average vacancy period increased with 60 days that was assumed to be the time employers need to place a vacancy and to train replacement of absent worker. In the advanced model, separate median wages and friction periods were used for occupational classes. Also, the length of vacancy chain in each occupational class was considered.^11^^,^^12^

All monetary values are presented in 2021 value, i.e. costs not available for 2021 were inflated to that year. We used the consumer price index as a converter.

### Statistical analyses

Multiple imputations were used to impute missing data for independent variables and covariates of planned analyses. The outcome variables, individual HCM and FCM values, were complete for all 10 650 subjects and were not imputed. Data were missing both due to loss of follow-up of subjects and due to missing measurements of available subjects. The overall amount of incomplete data was 28.8%. Variables used in multiple imputations are listed in the Supplementary table S3. Multiple imputations were conducted 10 times with the mice-function from the R package ‘mice’. Data were assumed to be missing at random. The pooled results were reported in the analyses. Complete case analyses are shown in the Supplementary Material.

Latent trajectories in the smoking data were identified by means of latent class growth modelling. Details of the procedure have been presented in the previous publication.^29^ In brief, the trajectory modelling was based on smoking status at each age between 5 and 47 years. Logistic models with one to six trajectory classes were tested. The Bayesian and Akaike information criteria, the Bayes factor and its log form, posterior membership probabilities, and absolute and relative class sizes were used to deduce that the model with six classes showed the best fit. Each subject was then assigned to the trajectory class with the highest posterior membership probability.

The distribution of productivity costs was zero-inflated and positively skewed among those who had them. A two-part regression model was used to account for the large proportion of subjects with estimated productivity costs equal to zero, and the positive skew of costs. Two-part models are commonly used for expenditure and productivity models with excess zeros. In the first part of the two-part model, we used a multivariable logit model predicting the probability that a subject would have productivity costs. In the second-part, productivity costs conditional on having any positive productivity costs were estimated using a generalized linear model (GLM) with logarithmic transformation on the costs. Both parts of the two-part model included a smoking variable of interest, i.e. either smoking trajectories or pack-years. The analyses were adjusted with weekly alcohol use, BMI, physical activity, hypertension and hypercholesterolemia, and conducted separately for women and men. The formula of the regression model was

$$\mathrm{Productiv}ity\;\mathrm{costs}\;∼\;\mathrm{Smoking}\;+\;\mathrm{Weekly}\;\mathrm{alcohol}\;\mathrm{use}\;+\;W{\mathrm{eekly}\;\mathrm{alcohol}\;\mathrm{use}}^{2}\;+\;\mathrm{BMI}\;+\;{\mathrm{BMI}}^{2}\;+\;\mathrm{Physical}\;\mathrm{Activity}\;+\;{\mathrm{Physical}\;\mathrm{Activity}}^{2}\;+\;\mathrm{Hypertension}\;+\;\mathrm{Hypercholestero}l\mathrm{emia}.$$

All analyses were done in R version 4.0.3 (2020-10-10).

## Results

### Characteristics of sample

The study sample consisted of 10 650 persons of which 49.5% were women (table 1). Of the total sample, 38.9% were never-smokers, 11.9% youth smokers, 10.8% young adult quitters, 11.8% late adult quitters, 4.8% late starters and 21.7% lifetime smokers. The mean pack-years among those who had ever smoked (i.e. had pack-years > 0) were 16.5 (SD 13.2) by age of 46, being the highest among lifetime smokers.

**Table 1:** Characteristics of sample

	Total (*N* = 10 650)	Never-smokers (*N* ∼ 4141)	Youth smokers (*N* ∼ 1269)	Young adult quitters (*N* ∼ 1155)	Late adult quitters (*N* ∼ 1260)	Late starters (*N* ∼ 512)	Lifetime smokers (*N* ∼ 2313)	*P*-value
Female sex	49.5%	54.0%	55.2%	44.5%	42.4%	44.0%	45.9%	<0.001
Educational level								<0.001
Basic	9.8%	6.4%	9.1%	9.0%	13.5%	7.4%	15.0%	
Secondary	65.7%	61.9%	65.5%	66.3%	70.8%	71.0%	68.0%	
Tertiary	24.5%	31.7%	25.4%	24.7%	15.6%	21.7%	16.9%	
Hypertension	49.1%	46.6%	44.6%	50.2%	54.2%	52.7%	51.8%	<0.001
Hypercholesterolemia	77.8%	76.3%	76.3%	80.2%	81.0%	78.8%	78.0%	<0.001
Physical activity	110.3 (119.3)	121.8 (122.8)	122.9 (124.6)	108.5 (115.6)	108.1 (117.9)	93.3 (112.6)	89.0 (110.0)	<0.001
BMI	27.0 (5.0)	26.7 (5.0)	27.1 (5.4)	27.2 (4.8)	28.1 (5.1)	27.4 (5.0)	26.9 (5.0)	<0.001
Weekly alcohol use	73.6 (123.3)	46.8 (74.9)	63.5 (98.6)	83.0 (129.9)	86.8 (128.1)	97.1 (168.7)	109.2 (166.7)	<0.001
Pack-years	7.7 (12.2)	0.6 (3.7)	3.9 (7.5)	9.1 (10.4)	12.5 (11.4)	11.7 (12.6)	17.9 (15.6)	<0.001
Pack-year categories								<0.001
Zero	53.4%	94.5%	52.4%	22.5%	8.6%	33.0%	25.6%	
Under five	10.0%	2.3%	23.6%	23.4%	20.2%	9.0%	4.5%	
Under 15	14.0%	1.6%	15.5%	30.5%	36.9%	21.1%	13.2%	
Under 30	14.4%	1.1%	6.5%	17.5%	25.4%	27.5%	31.5%	
Over 30	8.2%	0.5%	2.0%	6.0%	8.9%	9.3%	25.1%	
Wage	28 524 (18 416)	30 679 (19 829)	28 007 (17 441)	29 114 (17 844)	26 792 (16 151)	27 489 (18 181)	25 924 (17 340)	<0.001
Absence days	1726 (3400)	1381 (3124)	1626 (3322)	1688 (3419)	1976 (3618)	2099 (3830)	2185 (3601)	<0.001

Note: The table is based on pooled results of the imputed datasets. Numbers are shown as % or mean (SD).

BMI, body mass index.

The mean wage was different in smoking trajectory groups being lowest among lifetime smokers (25 924 euro; SD 17 340) and highest among never-smokers (30 679 euro; SD 19 829) (table 1). Similarly, mean number of sickness benefit days was highest among lifetime smokers (2185 days; SD 3601), and lowest among never-smokers (1381 days; SD 3124). Women had on average less sickness benefit days than men: mean 1497 (SD 3031) vs. 1951 (SD 3713) days (*P* < 0.001).

### Smoking and productivity costs

The mean lifetime productivity costs per one person of the total population were €95 375 (SD €185 730) estimated with HCM and €42 746 (SD €70 049) estimated with FCM. The mean productivity costs in smoking trajectories divided by sex and estimation method are shown in table 2. Both among women and men, the highest mean productivity costs were among lifetime smokers and lowest among never-smokers. Furthermore, the higher the number of pack-years, the higher the productivity costs. The same patterns were found both with HCM and FCM.

**Table 2:** Mean lifetime productivity costs per one person in smoking trajectories and pack-year classes estimated with advanced HCM and FCM

	Women					Men				
	% with costs	HCM €		FCM €		% with costs	HCM €		FCM €	
		**All**	**>0 costs**	**All**	**>0 costs**		**All**	**>0 costs**	**All**	**>0 costs**
Smoking trajectories										
Never-smokers	81	69 303	85 520	40 998	50 592	77	88 576	115 782	33 489	43 775
Youth smokers	86	83 071	96 172	48 117	55 706	79	96 556	122 637	34 471	43 781
Young adult quitters	85	73 450	86 023	43 234	50 635	82	107 293	131 644	35 543	43 610
Late adult quitters	85	93 605	110 438	48 784	57 557	84	116 667	139 627	39 582	47 372
Late starters	83	86 139	103 551	50 746	61 004	82	137 860	167 837	43 425	52 868
Lifetime smokers	85	90 390	105 777	55 159	64 549	85	144 773	171 083	50 675	59 884
Pack-years										
Zero	82	69 691	84 589	42 387	51 448	77	85 194	111 237	32 185	42 023
Under five	86	78 367	91 651	43 408	50 767	79	95 101	120 811	33 015	41 940
Under 15	87	88 965	102 772	51 960	60 023	81	116 367	142 937	38 341	47 096
Under 30	85	94 629	111 414	55 724	65 608	86	139 058	162 319	48 640	56 777
Over 30	81	133 615	164 235	62 305	76 583	89	184 427	206 513	60 765	68 042

Note: FCM, friction cost method; HCM, human capital method.

Table 3 shows the two-part regression results on association between smoking trajectories or pack-years and productivity costs by estimation method and by sex. Among women youth smoking, young adult quitting, late adult quitting and lifetime smoking associated with productivity costs compared with never-smoking. Among men, late adult quitting and lifetime smoking associated with productivity costs. Concerning cumulative smoking exposure, having smoked more than five pack-years associated with productivity costs both among women and men. All findings were independent of educational level, leisure-time physical activity, BMI, alcohol consumption, physical activity, hypertension and hypercholesterolemia. Results of complete case analyses (*n* = 4241) are shown in the Supplementary table S4.

**Table 3:** Results of two-part regression models on association of smoking trajectories and pack-year classes and productivity costs estimated with advanced HCM and FCM and stratified by sex

	Women				Men			
	HCM		FCM		HCM		FCM	
	exp(β) (95% CI)		exp(β) (95% CI)		exp(β) (95% CI)		exp(β) (95% CI)	
	Logit	LogOLS	Logit	LogOLS	Logit	LogOLS	Logit	LogOLS
Smoking trajectories								
Never-smokers	Ref	Ref	Ref	Ref	Ref	Ref	Ref	Ref
Youth smokers	1.51 (1.13–2.01)	1.18 (0.95–1.45)	2.26 (1.44–3.70)	1.06 (0.87–1.30)	1.12 (0.84–1.49)	1.07 (0.81–1.43)	1.05 (0.69–1.62)	0.78 (0.59–1.03)
Young adult quitters	1.41 (0.94–2.11)	1.01 (0.80–1.29)	1.67 (1.03–2.85)	0.92 (0.73–1.17)	1.31 (0.98–1.75)	1.12 (0.84–1.50)	1.34 (0.88–2.09)	0.89 (0.68–1.15)
Late adult quitters	1.30 (0.93–1.80)	1.28 (1.02–1.61)	1.77 (1.05–3.23)	1.08 (0.84–1.38)	1.49 (1.14–1.94)	1.42 (1.09–1.84)	1.42 (0.91–2.28)	0.94 (0.72–1.22)
Late starters	1.17 (0.71–1.91)	1.21 (0.87–1.67)	1.50 (0.75–3.44)	1.07 (0.76–1.53)	1.36 (0.90–2.05)	1.38 (0.95–2.00)	1.08 (0.58–2.15)	0.97 (0.64–1.48)
Lifetime smokers	1.45 (1.10–1.90)	1.42 (1.18–1.71)	1.80 (1.22–2.73)	1.35 (1.12–1.62)	1.62 (1.31–2.02)	1.88 (1.53–2.32)	1.56 (1.06–2.34)	1.43 (1.13–1.80)
Pack-years								
Zero	Ref	Ref	Ref	Ref	Ref	Ref	Ref	Ref
Under five	1.28 (0.97–1.68)	1.02 (0.84–1.25)	1.13 (0.74–1.79)	0.89 (0.71–1.11)	1.12 (0.86–1.45)	1.07 (0.81–1.41)	1.24 (0.79–2.01)	0.91 (0.68–1.22)
Under 15	1.40 (1.04–1.90)	1.29 (1.07–1.57)	1.66 (1.06–2.74)	1.12 (0.91–1.37)	1.31 (1.02–1.69)	1.42 (1.15–1.76)	1.32 (0.89–2.00)	1.02 (0.79–1.30)
Under 30	1.25 (0.92–1.70)	1.56 (1.24–1.97)	4.37 (2.07–11.27)	1.59 (1.26–2.00)	1.79 (1.41–2.27)	1.93 (1.61–2.32)	3.18 (1.85–5.89)	1.67 (1.30–2.14)
Over 30	1.00 (0.64–1.55)	2.82 (2.05–3.86)	8.60 (1.86–152.96)	2.16 (1.49–3.14)	2.49 (1.80–3.46)	2.90 (2.31–3.64)	4.11 (1.99–9.98)	1.86 (1.37–2.53)

Note: The two-part regression models were adjusted for educational level, leisure-time physical activity, BMI, alcohol consumption, hypertension, and hypercholesterolemia.

exp(β)=β-coefficient transformed with natural exponential function; FCM, friction cost method; HCM, human capital method; Logit, logistic regression model with logit link function; LogOLS, linear regression with ordinary least squares (OLS) and log-transformed-dependent variable.

## Discussion

Based on the highly accurate HCM and FCM approaches we show that continued smoking and higher smoked pack-years among adults associated with higher productivity costs. To our knowledge this is the first study to utilize mortality and work absenteeism data to calculate productivity costs for lifetime smoking. We used individual-level data on prospective birth cohort collected from questionnaires, clinical examinations and registers, combined with population-level data to estimate precise associations between smoking and productivity costs.

We found that among women smoking trajectories of youth smoking, young adult quitting, late adult quitting and lifetime smoking were associated with increased lifetime productivity costs compared with never-smokers. Among men late adult quitting and lifetime smoking were associated with increased productivity costs. Having smoked over five pack-years was associated with higher productivity costs in both sexes, and the higher the pack-years, the higher the costs. The associations between smoking and productivity costs were found both with advanced HCM and FCM, and the findings were independent from risky health behaviours and cardiometabolic health.

Our finding that the productivity costs were highest among lifetime smokers is in line with previous literature showing that smokers have higher productivity costs compared with former smokers.^31^ In our study, other trajectories than continuing smoking represented smoking histories where participants quit smoking during follow-up. Noteworthy, lifelong smoking trajectory was not only related to productivity costs, but also to highest number of smoked pack-years. As we found that higher number of pack-years associated with higher productivity costs, the association might be irrespective of the timing of smoking trajectory. We did separate analyses for smoking trajectories and pack-years.

Overall, our results suggest that accumulation of smoked pack-years might be more crucial to productivity costs than timing of smoking. However, further studies are needed to confirm if the associations are irrespective of age when smoking started as in our population most smokers started smoking around the same age. The trajectories mainly represented the ending age and the duration of smoking, and no comparisons between classes with same duration but different timing (i.e. different starting and ending age) of smoking could be made. Interestingly, previous literature suggests that productivity costs are similar among former smokers, independent of time since they quit.^31^ Given this previous finding, differences in productivity costs between the trajectories that were found in our study relate probably more to the duration of smoking.

The mechanisms by which smoking can associate with productivity costs are many. First, smoking is known to associate with poor health, mortality and work absence.^1^^,^^2^^,^^32^ However, smoking can affect also work outcomes negatively.^33^ Hiring a smoker is a risk for an employer,^34^ which can manifest as lower prospects for higher-wage or managing positions. However, in our advanced HCM and FCM models we used occupation-class specific wages as monetary value of production. Therefore, if smokers had lower-wage occupations, then the productivity costs would be lower than those of higher-wage non-smokers in case of similar work absence and mortality.

We found that differences in educational level, leisure-time physical activity, BMI, alcohol consumption, hypertension or hypercholesterolemia did not explain the association between smoking and productivity costs. Furthermore, the associations of smoking trajectories or cumulative exposure and productivity costs were mainly similar between women and men. This suggests smoking to have independent associations with productivity costs, and despite the sex.

### Strengths and limitations

The current study had several strengths. First, the lifelong approach to study the associations between smoking and productivity costs allowed us to explore not only the current smoking status, but also the timing, duration, intensity and cumulative exposure of smoking. Information on smoking was measured at several age-points with several characteristics. Second, a large and unselected population-based birth cohort was used in this study. Results of this naturalistic real-world data set are highly generalizable to general population in Western countries. The data collection started from the second trimester of cohort members’ antenatal period and follow-up lasted up to 50 years of age. The data collection was prospective reducing the potential for information bias, and questionnaire and clinical examination data were combined with comprehensive nationwide registers. Individual-level information on the outcome measures, i.e. sickness benefits, mortality and earnings were collected from nationwide registers that were complete and continuous without any loss of follow-up.

Third, multiple imputation was conducted for smoking variables and covariates to reduce selection bias and loss of statistical power due to missing data.^35^^,^^36^ The previous study from the same birth cohort data have shown that high alcohol consumption, low educational level, unemployment and being single at age 31 predicted lower participation at follow-up examination and questionnaires.^37^ This might have affected the results if the multiple imputations was not used. However, it should be noted that the selection of variables in multiple imputation model might also affect the results.

Fourth, individual-level data were combined with population-level data to estimate advanced HCM and FCM productivity costs. Our data allowed us to demonstrate more accurate estimates of productivity costs compared with the previous studies. Importantly, we were able to adjust HCM and FCM models for occupation-specific costs, macroeconomic conditions, vacancy chains and disability conditions. A specific methodological strength stems from the large availability of relevant and reliable Finnish register-based data. Especially, population-level data on occupational-specific vacancy durations and labor market conditions used in this study are unique within international literature.

The main limitation of this study was that the age intervals of follow-up questionnaires (14, 31 and 46 years) were rather long apart. Smoking status was asked only in these three time points and was estimated for the remaining age points that were used in smoking trajectory model based on starting and ending age (between 5 and 47 years). To reduce recall bias due to gaps between follow-up questionnaires, the 31 years follow-up assessment was selected as the primary source for data on smoking starting age and the 46 years follow-up for smoking ending age.

Second limitation is that the individual-level register data on work absence only included sick leaves longer than 10 days. Additionally, the previous study in Finland has shown that people not currently working because of unemployment or studying do not receive sickness allowance or disability pension, to which they are similarly entitled to, as easily as employed people.^38^ If this were the case, then the productivity costs could be higher than was found, as some smokers were not present in registers. Furthermore, the productivity cost estimates were restricted to lost paid employment. No value for non-market production, e.g. informal care and unpaid volunteer work, was estimated. This may have caused underestimation of productivity costs. Neither was the reduced productivity while at work, presenteeism, accounted for. This was inevitable because only objective register data were used in this study and no objective measures are available for presenteeism.

## Conclusion

In this large, population-based birth cohort study we found that lifetime smokers and those with highest smoked pack-years had the highest productivity costs. Other risky health behaviours, cardiometabolic health or sex did not explain the association. As cumulative exposure to smoking seems to be more crucial in relation to productivity costs than timing of smoking, preventive measures should be targeted to those with the highest pack-years or at risk to become tobacco dependent to avoid productivity costs.

## Supplementary Material

ckae057_Supplementary_Data

## Data Availability

Data are available from the Northern Finland Birth Cohort (NFBC) for researchers who meet the criteria for accessing confidential data. Please, contact NFBC project center (NFBCprojectcenter@oulu.fi) and visit the cohort website (www.oulu.fi/nfbc or http://urn.fi/urn:nbn:fi:att:bc1e5408-980e-4a62-b899-43bec3755243) for more information. Key points Lifetime smokers had the highest productivity costs followed by late starters, late adult quitters, young adult quitters, youth smokers and never-smokers. The higher the number of smoked pack-years, the higher the productivity costs. Other risky health behaviours or cardiometabolic health do not explain the association between smoking and productivity costs. The association of smoking and productivity costs depend on dose and the duration of smoking more than of age when smoking occurred.
