# Supplementary material for: Productivity costs of lifelong smoking—the Northern Finland Birth Cohort 1966 study
Source: Eur J Public Health. 2024 Mar 29;34(3):572–7. doi: 10.1093/eurpub/ckae057 (PMC11161164; doi:10.1093/eurpub/ckae057)
Supplement: ckae057_Supplementary_Data [file ckae057_supplementary_data.pdf]

Supplementary material

Supplementary Table 1. Collection of individual level data on covariates.

Variable	Source	Years when collected	Definition	Use	Type of variable	Coding
Educational level	Questionnaires	Ages 31 and 46 years	Educational level was asked at questionnaires as highest basic education, highest secondary, and highest tertiary education by age of 31 and 46.	Educational level at age 46	Ordinal	3 = tertiary education (> 12 years; university or university of applied sciences)<br>2 = secondary education (9-12 years; upper secondary school or vocational school)<br>1 = primary education (≤ 9 years; comprehensive school)
Physical activity	Questionnaires	Ages 14, 31, and 46 years	Leisure-time physical activity was asked as: ‘How often do you exercise on your leisure-time with high intensity’ with options ‘Once a month or less’, ‘2-3 times a month’, ‘Once a week’, ‘2-3 times a week’, ‘4-6 times a week’ and ‘daily’, and ‘What is the duration of each exercise with high intensity’ with options ‘none’, ‘less than 20 minutes’, ’20-39 minutes’, ‘40-59 minutes’, ‘1-1.5 hours’ and ‘more than 1.5 hours’51. By multiplying the frequency and duration, weekly minutes of moderate-to-vigorous intensity physical activity (MVPA) was obtained and further divided into physically active (MVPA≥150min/week) and physically inactive (MVPA<150min/week) groups, according to the current physical activity recommendations for health.	Physical activity as hours/month at age 46.	Continuous	Hours per month

BMI	Clinical examinations and questionnaires	Ages 14, 31, and 46 years	Weight and height were measured in clinical examination and asked with the postal questionnaire. BMI (kg/m^2) was calculated from weight and height using the measured values as primary source.	BMI as kg/m^2 at age 46	Continuous	Kg/m^2
Alcohol use and consumption	Questionnaires	Ages 14, 31, and 46 years	Alcohol consumption at age 14 was assessed with the question 'Alcohol consumption (beer or other alcoholic drinks)' with the options 'I have never drunk', 'I have tasted once', 'I have drunk a few times', 'I drink monthly' and 'I drink weekly'. At age 14 subjects who had drunk alcohol a few times or more were categorized as users. At age 31 and 46 the use of alcohol beverages was asked according to type of beverage and how often usually a person uses that and what is a typical number of portions when used. Daily mean alcohol consumption was calculated for ages 31 and 46 as g/day using information on typical portion sizes and alcohol content in Finland.	Alcohol consumption as g/day at age 46	Continuous	Grams per day
Presence of hypertension	Clinical examinations, questionnaires, and registers	Ages 31 and 46 years	Hypertension was defined as having a mean of measured systolic blood pressure ≥ 140 mmHg, diastolic blood pressure ≥ 90 mmHg, self-reported diagnosis of hypertension, or using antihypertensive medication (ATC codes C02 antihypertensives, C03 diuretics, C07 beta blocking agents, C08 calcium channel blockers, and C09 agents acting on the renin-angiotensin system) according to national register of medication reimbursement.	Hypertension at age 46	Categorical	1 = hypertension<br>0 = no hypertension

Presence of hypercholesterolemia	Clinical examinations and registers	Ages 31 and 46 years	The presence of hypercholesterolemia was noted in case of triglyceride level > 2.0 mmol/l, LDL-cholesterol > 3.0 mmol/l, HDL cholesterol < 1.0 mmol/l, or in case of current lipid-lowering therapy (ATC code C10 lipid modifying agents) in register.	Hypercholesterolemia at age 46	Categorical	1 = hypercholesterolemia<br>0 = no hypercholesterolemia

BMI= body mass index, ATC= Anatomical Therapeutic Chemical, LDL= Low-density lipoprotein cholesterol, HDL= High-density lipoprotein cholesterol.

Supplemental Table 2. Collection of population level data used in HCM and FCM

Variable	Available data	Used for	Calculated as	Data level	Data source	Available	Other notes
GDP growth rate	Annual GPD growth	Proxy for annual wage growth before 1995 and after 2016	n.a.	Population level data	World Development Indicators, The World Bank and Statistics Finland	1961-2021	Annual 1.59% growth rate (mean of 2016-2021) used after 2021
Occupational class	Annual occupational class	Occupation specific calculations	n.a.	Individual level data used to calculate occupation specific wages and friction periods	Statistics Finland	1995, 2000, 2004-2018	1995 value used for 1984-1999, 2000 value used for 2000-2003, 2018 value used for 2019-2031
Disability free life expectancy	Disability pensions	Adjusted HCM	% of same aged population on disability pension	Population level data	Finnish Institute for Health and Welfare	1996-2021	Data available for age group 25-64 was used in calculations
Unemployment rate (u)	Annual % employed for both genders	Adjusted HCM and LVC calculations	1- %employed	Population level data	Statistics Finland	1997-2020	Data was extended by using the mean of first/last five observations of the relevant age group
Life expectancy	For both genders and ages between 18-65.	PLDF (disability free life expectancy / life expectancy)	Estimated after 2021 by the projection in year 2021 for ages 55-56, 2025 for ages 57-61, 2030 for ages 62-65	Population level data	Statistics Finland	1986-2021, projection for 2021, 2025, 2030, 2035	Expanded by using the first observation for appropriate age. Projection based on 2021 prediction.
Proportion of employed jobseekers (α)	Jobseekers, unemployed jobseekers; separately for	LVC calculations	Number of jobseekers minus number of unemployed jobseekers,	Population level data	Statistics Finland	2006-2021	Data extended by using the mean of 5 first/last observations each gender and

	occupational classes		divided by number of jobseekers				occupational group, using the appropriate age group.
Work ability (WA)	Self-reported work ability	Adjusted HCM	Mean of individual answers in each gender and occupation specific group	Individual level data used to calculate population level index	Questionnaires to NFBC1966 cohort: "How would you score your current work ability as compared to lifetime best?" scaled from 0 (no work ability) to 10 (at maximum)	1997 and 2012	One occupational class and gender specific index was used for 1997 and 2012
Work ability decline (WAD)	WA from NFBC1966 questionnaires	Adjusted HCM	1 - (mean(WA in year 1997) – mean(WA in year 2012)) / (mean(WA in year 1997))	Individual level data used to calculate population level index	Questionnaires to NFBC1966 cohort	1997 and 2012	
Occupational vacancy period	Annual average vacancy of filled positions for each occupational class	Occupation specific and adjusted FCM	n.a.	Population level data	Ministry of Economic Affairs and Employment and Statistics Finland	2006-2021	Average of 2006-2009 used as an estimate for 1984-2005, and average of 2018-2021 used as an estimate for 2022 onwards
Occupational friction period	Average occupation-specific vacancy period increased with 60 days	Occupation specific and adjusted FCM	Occupational vacancy period + 60 days	Population level data	Ministry of Economic Affairs and Employment and Statistics Finland	1984 onwards	
Length of vacancy chain (LVC)	α and u presented previously	Adjusted FCM	$\alpha * (1- u) / u$	Population level data	Statistics Finland	1984 onwards	

ICD = International Classification of Diseases, SII = Social Insurance Institution, FCP = Finnish Center for Pensions, GDP = Gross Domestic Product, n.a. = not applicable.
Used links:

Statistics Finland. https://www.stat.fi/index_en.html
Social Insurance Institution of Finland. https://www.kela.fi/web/en
Finnish Centre for Pensions. https://www.etk.fi/en/
Finnish Tax Administration. https://www.vero.fi/en
Statistics Finland. Monetary value factor. https://dev.verkko.stat.fi/til/khi/2021/khi_2021_2022-01-14_tau_001.html
Statistics Finland. Monetary value factor. https://www.stat.fi/tup/laskurit/rahanarvonmuunnin_en.html
World Development Indicators. The World Bank. https://data.worldbank.org/indicator/NY.GDP.MKTP.KD.ZG
Statistics Finland. https://www.tilastokeskus.fi/fi/luokitukset/ammatti/
Finnish Institute for Health and Welfare. https://sotkanet.fi/sotkanet/en/metadata/indicators/306
Finnish Institute for Health and Welfare
https://sotkanet.fi/sotkanet/fi/taulukko/?indicator=szbxt64wAwA=&region=szYPiTeyNioAAA==&year=sy5zsi7T0zUEAA==&gender=m;f;t&abs=f&color=f&buildVersion=3.0
Statistics Finland. https://stat.fi/tilasto/tyti
Statistics Finland. https://pxdata.stat.fi/PxWeb/pxweb/fi/StatFin_Passiivi/StatFin_Passiivi__tyti/statfinpas_tyti_pxt_11pp_2020.px/
Statistics Finland. https://stat.fi/tilasto/kuol
Statistics Finland. https://statfin.stat.fi/PxWeb/pxweb/fi/StatFin/StatFin__kuol/statfin_kuol_pxt_12ap.px/
Statistics Finland. https://stat.fi/tilasto/vaenn
Statistics Finland. https://statfin.stat.fi/PxWeb/pxweb/fi/StatFin/StatFin__vaenn/statfin_vaenn_pxt_139l.px/
Statistics Finland. https://statfin.stat.fi/PxWeb/pxweb/fi/StatFin/StatFin__tyonv/statfin_tyonv_pxt_12v6.px/table/tableViewLayout1/
Statistics Finland. https://stat.fi/en/statistics/tyonv
Statistics Finland. https://statfin.stat.fi/PxWeb/pxweb/fi/StatFin/StatFin__tyonv/statfin_tyonv_pxt_12v5.px/table/tableViewLayout2/

Supplemental Table 3. Variables used in the multiple imputation procedure.

	% of missing data
**Predictors in regression model**	
Smoking trajectories	47.24
Pack-years by age 46	25.82
**Covariates**	
Sex	0
Alcohol consumption at age 46	40.29
BMI at age 46	37.97
Hypertension at age 46	47.58
Hypercholesterolemia at age 46	48.89
Physical activity at age 46	41.52
Educational level by age 46	40.33
**Outcome variables**	
HCM productivity costs	0
FCM productivity costs	0
**Other variables**	
Smoking status at age 14	0.64
Smoking status at age 31	24.95
Smoking status at age 46	40.98
Pack-years by age 31	32.16
Mother's smoking during pregnancy	2.27
Father's smoking during adolescence	5.15
Passive smoking at age 31	25.87
Passive smoking at age 46	41.44
Alcohol use at age 14	0.7
Alcohol consumption at age 31	24.6
BMI at age 14	7.4
BMI at age 31	24.58
Self-reported education by age 31	33.78

Hypertension at age 31	48.25
Hypercholesterolemia at age 31	48.48
Physical activity at age 31	25.11
Use of other drugs at age 14	0.75
Problems with alcohol at age 31	24.58
Problems with alcohol at age 46	40.72
Problems with other intoxicants at age 31	24.65
Problems with other intoxicants at age 46	40.78
Circumference of pelvis at age 31	49.75
Circumference of waist at age 31	49.75
Circumference of pelvis at age 46	49.24
Circumference of waist at age 46	49.2
Diagnosis of any mental disorder	44.17
Mean wage	5.89

Supplemental Table 4. Complete case results of two-part regression models on association of smoking trajectories and pack year classes and productivity costs estimated with advanced HCM and FCM and stratified by sex.

	Women				Men			
	HCM		FCM		HCM		FCM	
	exp(β) (95% CI)		exp(β) (95% CI)		exp(β) (95% CI)		exp(β) (95% CI)	
	Logit	LogOLS	Logit	LogOLS	Logit	LogOLS	Logit	LogOLS
**Smoking trajectories**								
Never-smokers	Ref	Ref	Ref	Ref	Ref	Ref	Ref	Ref
Youth smokers	1.18 (0.95-1.45)	1.10 (0.89-1.35)	1.06 (0.87-1.30)	1.07 (0.90-1.29)	1.07 (0.81-1.43)	1.06 (0.81-1.38)	0.78 (0.59-1.03)	0.84 (0.66-1.07)
Young adult quitters	1.01 (0.80-1.29)	0.98 (0.73-1.32)	0.92 (0.73-1.17)	0.93 (0.76-1.16)	1.12 (0.84-1.50)	1.12 (0.90-1.38)	0.89 (0.68-1.15)	0.83 (0.66-1.05)
Late adult quitters	1.28 (1.02-1.61)	1.25 (1.00-1.57)	1.08 (0.84-1.38)	1.08 (0.87-1.35)	1.42 (1.09-1.84)	1.17 (0.93-1.47)	0.94 (0.72-1.22)	0.78 (0.62-0.99)
Late starters	1.21 (0.87-1.67)	1.15 (0.82-1.62)	1.07 (0.76-1.53)	1.15 (0.85-1.59)	1.38 (0.95-2.00)	1.36 (0.99-1.86)	0.97 (0.64-1.48)	1.06 (0.75-1.55)
Lifetime smokers	1.42 (1.18-1.71)	1.19 (0.99-1.42)	1.35 (1.12-1.62)	1.22 (1.03-1.44)	1.88 (1.53-2.32)	1.40 (1.15-1.70)	1.43 (1.13-1.80)	1.15 (0.94-1.41)
**Pack years**								
zero	Ref	Ref	Ref	Ref	Ref	Ref	Ref	Ref
under five	1.02 (0.84-1.25)	1.05 (0.84-1.31)	0.89 (0.71-1.11)	0.85 (0.69-1.04)	1.07 (0.81-1.41)	1.08 (0.86-1.37)	0.91 (0.68-1.22)	1.06 (0.83-1.39)
under 15	1.29 (1.07-1.57)	1.19 (0.98-1.45)	1.12 (0.91-1.37)	1.09 (0.90-1.31)	1.42 (1.15-1.76)	1.27 (1.06-1.54)	1.02 (0.79-1.30)	0.92 (0.74-1.14)
under 30	1.56 (1.24-1.97)	1.27 (1.04-1.54)	1.59 (1.26-2.00)	1.28 (1.04-1.59)	1.93 (1.61-2.32)	1.43 (1.21-1.68)	1.67 (1.30-2.14)	1.38 (1.11-1.72)
over 30	2.82 (2.05-3.86)	1.82 (1.35-2.46)	2.16 (1.49-3.14)	1.67 (1.20-2.40)	2.90 (2.31-3.64)	1.75 (1.43-2.15)	1.86 (1.37-2.53)	1.47 (1.13-1.94)

The two-part regression models were adjusted for educational level, leisure-time physical activity, BMI, alcohol consumption, hypertension, and hypercholesterolemia. HCM = human capital method, FCM = friction cost method, exp(β) = β-coefficient transformed with natural exponential function, Logit = Logistic regression model with logit link function, LogOLS = Linear regression with Ordinary least squares (OLS) and log-transformed dependent variable.
